# Surgical Reconstruction of Nonunion after Iatrogenic Scarf Osteotomy

**DOI:** 10.3390/ijerph18115620

**Published:** 2021-05-25

**Authors:** Mercedes Ortiz-Romero, Luke D. Cicchinelli, Álvaro Fernández-Garzón, Luis M. Gordillo-Fernández

**Affiliations:** 1 Department of Podiatry, University of Seville, 41009 Seville, Spain; mortizrom@gmail.com; 2 Independent Researcher, Philadelphia, PA 19107, USA; 3 Independent Researcher, 11201 Algeciras, Spain; alvarofdezgarzon@gmail.com

**Keywords:** scarf, iatrogenic, nonunion, autograft, surgery, foot, orthopedics, foot pathologies, walking aid device

## Abstract

We present the case of a young patient, 32 years old, with nonunion in the diaphysis of the first metatarsal after scarf osteotomy for correction of hallux valgus. After removal of the failed osteosynthesis material and preparation of the bone fragments, a calcaneal bone autograft, previously extracted from the patient, was placed in the nonunion area. The new physiological position of the first metatarsal in the three planes was checked intraoperatively, and autograft and fragment fixation was performed using a combination of a low-profile plate with six screws and two interfragmentary screws. The advantage of using an autogenous graft is that it provides corticocancellous bone and great osteogenic capacity with little antigenic capacity. This makes it an excellent option in many situations in foot and ankle surgery. Regarding the fixation method, we used the two most commonly used techniques for osteosynthesis of bone grafts in cases of bone nonunion, combining plates with locking screws and two interfragmentary screws. This provides greater stability of the bone fragments in the three planes and makes it possible to bring forward when the patient starts postsurgical loading.

## 1. Introduction

Hallux valgus (HV) is a common deformity with a prevalence of 23% in people between 18 and 65 years of age, increasing to 36% in people over 65 years of age [1]. Numerous procedures have been described for the correction of hallux valgus deformity, and many factors must be taken into account when deciding which surgical procedure to perform. 

Numerous surgical techniques have been described for the correction of HV deformity, with reports of good postoperative results, by both open surgery [2] and minimally invasive surgery [2,3,4,5,6].

The literature supports the use of open techniques for severe HV deformities [2,5] based on the possibility of using osteosynthesis material; however, there are currently new osteosynthesis techniques, such as the minimally invasive intramedullary nail device (MIIND) [3], which allow severe deformities to be corrected with satisfactory results.

In the present case, it could be deduced that an open scarf technique had been performed. Scarf Z-osteotomy is a versatile technique with satisfactory results, as can be seen in [3], although it has a variable rate of complications [7,8,9,10,11,12]. These include delayed wound healing, pain caused by metatarsalgia, skin irritation over a small protrusion of the screw, osteoarthritis of the joint, “troughing” of the metatarsal with loss of weight, and proximal metatarsal fracture [8,9,10,11,12].

In the case presented below, the main complication was due to the nonunion of an osteotomy performed as a consequence of a stress fracture in the proximal area of the first metatarsal, causing excessive shortening of the first radius as well as a pathological position in adduction and plantar flexion. Proximal fracture of the first metatarsal secondary to scarf osteotomy is not a frequent complication and may be associated with an error of fixation with the osteosynthesis material and with poor patient compliance [10].

## 2. Case Report

We present the case of a 32-year-old female patient who was consulted due to severe pain in the first metatarsal of the right foot after previous surgery for hallux valgus using the scarf technique, fixed with two 3 mm cannulated screws.

**Figure 1:**
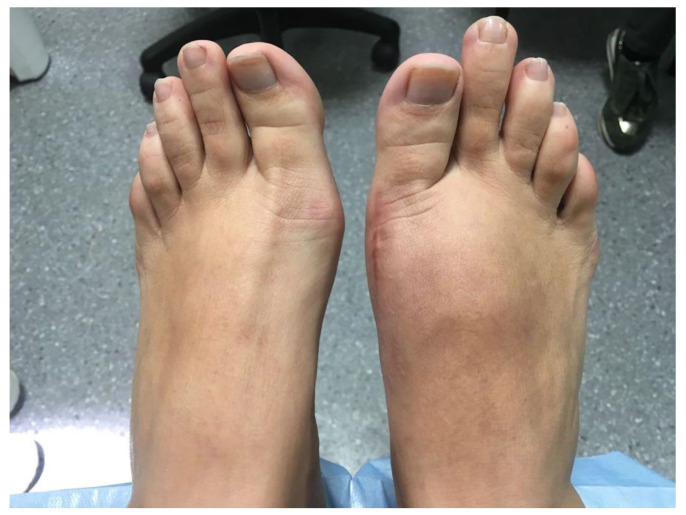
Image of appearance of both feet on arrival at clinic, highlighting shortening of first radius of the right foot with respect to the left foot.

Clinical examination revealed a cavus valgus foot with persistent edema and erythema evolved over 2 years. The shortening of the first radius in the operated foot with respect to the contralateral foot was significant, as was a hypertrophic and sensitive scar (Figure 1).

**Figure 2:**
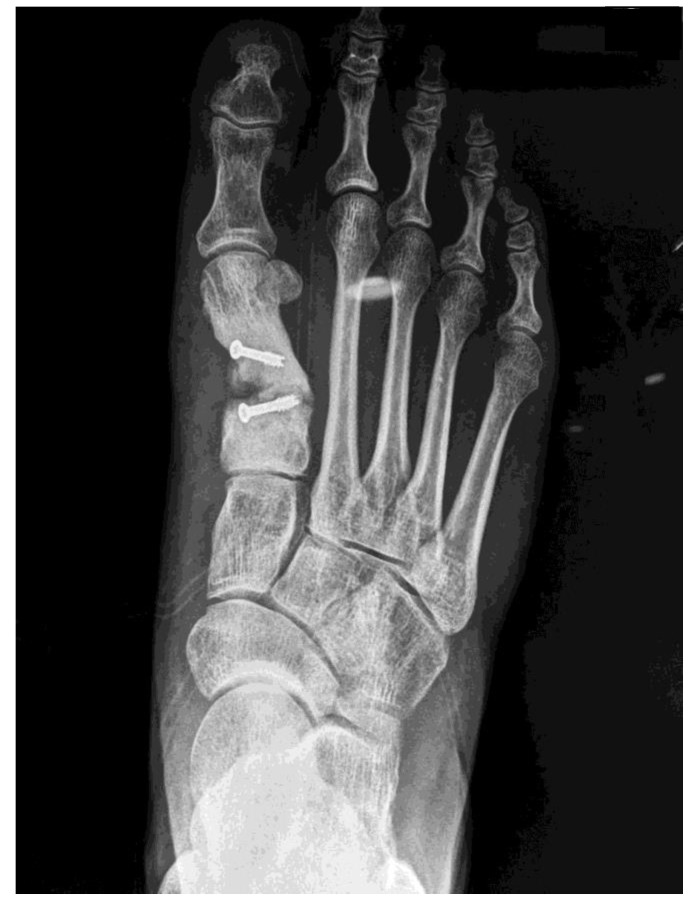
Rx AP immediately preoperative.

**Figure 3:**
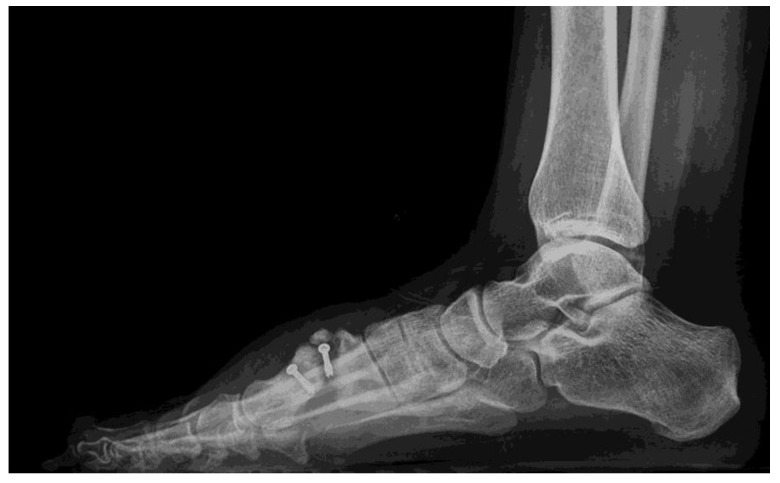
Rx Lateral immediately preoperative.

Finally, the radiological examination showed a complete transverse fracture with a dorsal free fragment coinciding with the proximal screw of what appeared to be a scarf Z-osteotomy. After the fracture, severe shortening of the first radius, medial displacement of the distal fragment of the metatarsal, and rotation associated with plantar flexion were observed (Figure 2 and Figure 3).

The patient’s medical examination revealed no systemic, cardiac, neurological, or rheumatological diseases. 

### 2.1. Surgical Procedure

The patient received a spinal block with 0.75% bupivacaine. A tourniquet was placed 10 cm below the fibular head with a pressure of 250 mm Hg.

First, an oblique incision was made in the lateral side of the calcaneus for extraction of a 2 × 2 cm bone graft in accordance with a technique described by Mahan [13]. 

Second, a double semi-elliptical incision was made over the previous scar, from the medial area of the first cuneometatarsal joint to the base of the proximal phalanx of the first toe, for the approach to and exposure of the first radius. This was to ensure that the old, sensitive, painful scar was eliminated and to create a new, more physiological scar.

The old and failed osteosynthesis material was removed. The calcaneal bone graft was placed in the nonunion area. At this time, the physiological position of the first metatarsal was sought by means of an evaluation in the three planes and by simulating the load. We consider this to be the most important surgical step, since the proper functionality of the first radius in the long term depended on this action.

It is important to highlight that the graft was carved manually with an oscillating saw prior to the operation, creating a medial and plantar base wedge that would help to correct the pathological position of the first metatarsal once it was in place.

The fragments were then fixed by placing a low-profile T-06 Arthrex osteosynthesis plate set with six locking screws. This was reinforced with the implantation of two interfragmentary screws placed obliquely. 

Then 2/0 synthetic absorbable suture was used to close the joint capsule in the first radius, 3/0 synthetic absorbable suture to close the deep fascia in the first radius and calcaneal graft extraction area, and Biosyn 4/0 to close the skin with continuous suture.

### 2.2. Postsurgical Procedure and Evolution

The patient remained immobilized for 8 weeks and then began partial weight-bearing with the aid of a walker boot for a further 4 weeks.

After 12 weeks, the patient had no pain or limitations and began to wear physiological footwear. At 6 months, she began to practice running, and the metatarsal adequately accepted the ground reaction force. The patient is currently preparing for physical tests for entry into the Spanish Army.

At 14 months post-surgery, we performed a small surgical intervention to remove the two interfragmentary screws, which were causing skin irritation.

### 2.3. Results

The post-surgical radiological images show good positioning of the first radius in the different planes, an increase in length achieved with the graft, and the two fixation systems used to provide greater stability to the bone fragments. Additionally visible is the bone defect at the level of the lateral aspect of the calcaneus after removal of the autogenous graft. This will be filled with new bone over the next few months until a calcaneus with normal morphology is achieved.

**Figure 4:**
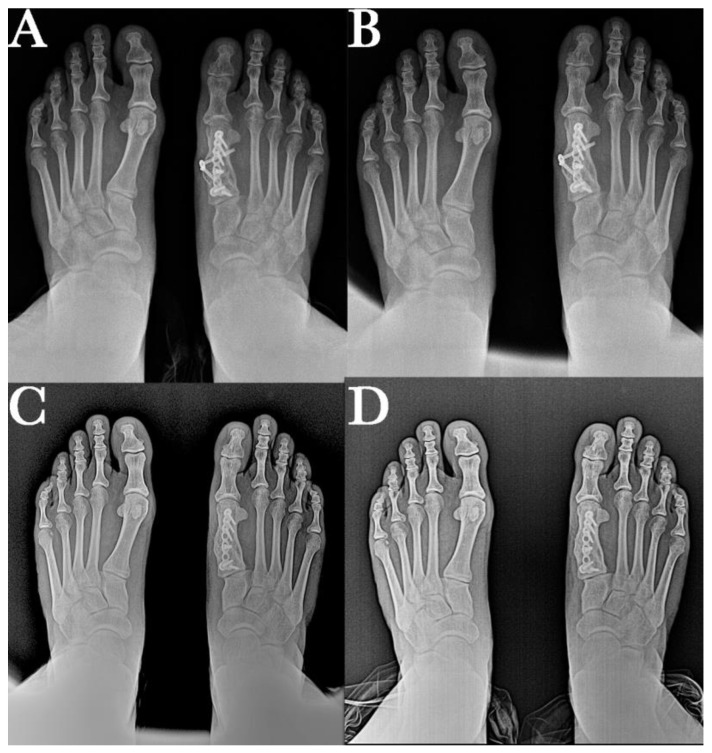
Correct position of the first metatarsal in the sagittal plane at (**A**) 6-month, (**B**) 12-month, (**C**) 18-month, and (**D**) and final 24-month follow-up after surgery, showing bone callus consolidation and remodeling, and maintaining correction of the analyzed radiographic parameters.

**Figure 5:**
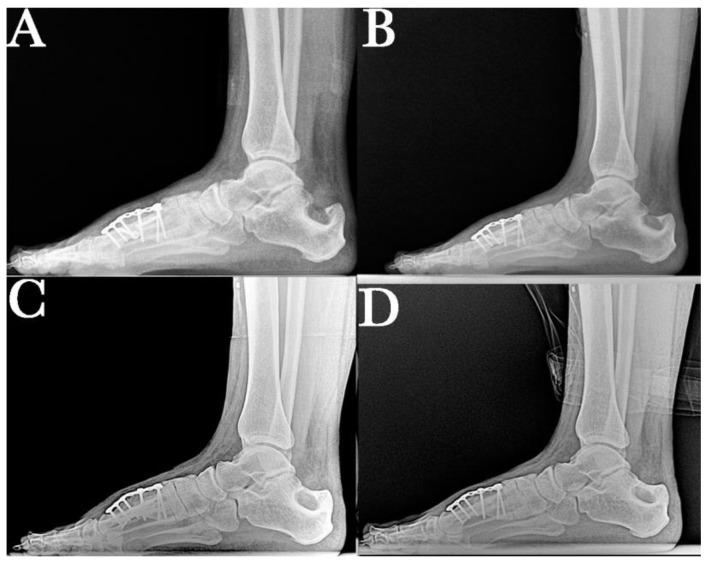
Correct position of the first metatarsal in the transverse plane and the evolution of the osseointegration process of an autologous graft. Images at (**A**) 6-month, (**B**) 12-month, (**C**) 18-month, and (**D**) final 24-month follow-up after surgery, showing bone callus consolidation and remodeling, and maintaining correction of the analyzed radiographic parameters.

Anteroposterior and lateral control radiographs were taken at 6, 12, 18, and 24 months. This radiological study shows the consolidation and position of the first radius as well as the remodeling process that occurs in the calcaneus after autograft removal (Figure 4 and Figure 5).

## 3. Discussion

Due to its versatility, the scarf Z-osteotomy is a widely used surgical technique. This technique requires a lot of skill and presents a steep learning curve. To avoid complications, it should be performed by a well-trained surgeon with in-depth knowledge of the principles of osteosynthesis [1,7,8,9,10,11,12].

Proximal fracture of the first metatarsal after the procedure is described as a possible complication; however, the percentages in the literature are very low [8,11]. Coetzee reported that the most frequent complication of this procedure is the "troughing" effect (35%) and that proximal fracture was observed in 2 of 20 cases studied (10%) [8]. Another study, by Misket et al., reported that, of 70 cases operated on by scarf osteotomy, only 2 patients (2.8%) suffered stress fractures in the proximal zone of the first metatarsal [11]. Kim et al. studied 115 patients undergoing surgery and found four cases of fracture of the first metatarsal, representing 3.5% of the cases [9]. More recently, Lenz et al. studied the possible complications present in 106 patients (118 feet) operated on for hallux valgus using the scarf technique. They found complications in 12 patients (10.2%) who underwent the operation, but only one had a fracture of the first metatarsal (0.8%) [10]. Finally, in a 3-year follow-up study of 166 patients, Rajeev and Turnia described three cases of osteochondritis but no cases of fracture of the first metatarsal (0%) [12].

Additionally, we reviewed the existing literature regarding the use of bone grafts to fill existing defects or correct postsurgical fractures or bone nonunions. Autologous bone grafts, allogenic bone grafts, synthetic bone graft substitutes, and orthobiologics are often used for such purposes [14,15,16,17,18,19]. While synthetic bone substitutes and growth factors lack osteoinductive capacity and allogeneic bone grafting lacks osteogenic potential, autologous bone grafting has become the gold standard and the choice of the vast majority of surgeons [14,15,16]. With osteoconductive, osteoinductive and osteogenic properties, autologous bone grafts integrate well into the host bone [14]. Furthermore, autografts are 100% histocompatible with no risk of disease transmission [15]. A meta-analysis by Lareau et al. including 159 studies (5327 patients) revealed that the success of autografting for bone repair in foot and ankle surgery was superior (95.1%) compared to the use of structural allografts (86.9%) [16].

Foot and ankle surgeons have several options when it comes to harvesting autologous bone grafts. The iliac crest has been the common donor site and is still frequently used. However, sufficient bone can also be expeditiously harvested from the proximal and distal tibial metaphyses, the calcaneus, and the intramedullary canals of both the tibia and femur. All of these donor sites have been shown to have high concentrations of mesenchymal stem osteoprogenitor cells [15]. The size of the graft needed and the anatomic proximity make the calcaneus an easily accessible site for harvesting autografts for foot and ankle procedures [17,18,19,20].

Complications in patients who have undergone calcaneal bone grafting have also been the subject of study. O’Malley et al. investigated the possible postoperative complications of 393 patients who had a calcaneal graft removed; 86.2% did not report any problems. Most of the complications that occurred were minor and related to discomfort at the incision site. Only three patients (1.4%) had more significant complications (one graft fracture, one calcaneal stress fracture, and one sural nerve injury) [17]. Finally, Cross and DiDomenico conducted a retrospective study of 242 patients (247 procedures) who underwent calcaneal graft harvesting, noting complications in only 6 cases. Of these, five (2.02%) presented neuritis of the sural nerve and one (0.41%) presented hypertrophic scarring of the donor site [21]. These results indicate that the calcaneus is a reliable, easy-to-access, and safe route for harvesting an autogenous bone graft, with a low incidence of complications in foot and ankle surgery [15,22]. 

With respect to the fixation technique for fractures, although intramedullary screw fixation results in a high union rate, delayed healing and complications can occur [23]. Therefore, in recent years, many surgeons have preferred to use titanium plates and autografts to stabilize old fractures in the metatarsals [24] and to repair anatomical defects in the area as a consequence of trauma or tumor excision [25,26]. Titanium locking plate implants provide long-term stability and resistance to stress and strain in the forefoot. This is particularly important because of the workload on the first metatarsal during the gait cycle [27]. Saxena et al. [28] compared fixation using two oblique interfragmentary screws with fixation using a locking plate and concluded that the locking plate is a good osteosynthesis technique that allows for an earlier postoperative loading time.

Brissey [29] studied the postoperative course of 49 patients who underwent 53 TMT1 arthrodeses for hallux valgus intervention. The author noted that locking plates combined with a compression screw presented a favorable tension-side implant location that closed the fusion site under load. In a recent study, Buda et al. [30] compared the nonunion complications in 88 patients (189 joints) who underwent first cuneometatarsal joint fusion. The fixations were made using three different fastening systems: with a plate only, with screws only, and with hybrid construction using plate and screws. Ten patients (11.4%) developed nonunion involving a total of 17 TMT joints. Of these TMT joints, 11 (16.4%) were fixed with a plate system, 5 (4.8%) were fixed with screws only, and 1 (5.9%) was fixed with the hybrid construction. In our case, we also used both techniques to favor the stability and viability of the implanted autogenous graft in all three planes when the first metatarsal is loaded.

## 4. Conclusions 

Nonunion as a consequence of a proximal fracture of the first metatarsal is a rare but very painful complication after hallux valgus surgery using the scarf technique. The use of autologous bone graft to repair the defect and to achieve the proper position of the first metatarsal was successful in the case presented here. The autologous bone graft has integrated well into the host bone thanks to its osteoconductive, osteoinductive, and osteogenic properties.

For harvesting an autogenous bone graft, the calcaneus can be a reliable, easy-to-access, and safe route, with a low incidence of complications in foot and ankle surgery. The use of the hybrid fixation system to resolve this case, with a combination of a locking plate and two oblique interfragmentary screws, provided reliable fixation allowing for correct osseointegration of the autograft and the necessary stability of the first metatarsal against ground reaction forces during the gait cycle.

## Data Availability

Not applicable.
